# Brevities

**Published:** 1848-01

**Authors:** E. Taylor


### EXPOLIATION FROM TOO HARD PRESSURE OF PLATE.

Does F. H. C., who reports case No. 1, for the October No. of the Dental Register, believe "that the pressure of a well fitted plate, would produce expoliation in a 'few days.'" We have seen small expoliations resulting from the developement of alveolar abscess on the palital surface, where the pressure from plate had not existed. E. T.

### TEETH HOLDERS.

We had the pleasure of introducing to the profession some instruments for the purpose of holding teeth, while grinding. They are made of two pieces of thin steel plate, of different widths to suit the width of the teeth. They are riveted together at the end which is inserted into the handle, and stand a half inch or so apart at the other end, and are drawn and held together on the tooth by a slide; which ends are fitted to the front and rivet sides of the tooth; for perpendicular rivets there is a hole very near the end, and a little distance further, a slit; the hole receives the rivet nearest the base; the corners of the plate are rounded so as to be out of the way. For the transverse rivets, the hole is near the corner, and the slit tranverse. The one for pivot teeth is made to embrace the cutting end, and the tooth being wider at that end, is not easily drawn out, and being thicker at the base, is not pushed in by grinding on the base. E. T.

To make Adhesive Wax for holding the Teeth while articulating.—Take common Beeswax, 1 pound, Gum Mastic 2 ounces, Whiting 1 ounce, and if for winter use, Tallow 1 ounce. Melt the wax and stir in the Mastic and Whiting; after pulverizing them, keep well stirred, until it begins to cool, then pour into shallow vessels. By heating the plate and teeth a little before applying them, this wax adheres very tenaciously, and is not liable to be dropping off, when they get wet in the mouth.

From Dr. S. Griffith, Louisville, Ky.

To cut off the crowns of Teeth, preparatory to inserting on Pivots.—We now use the finest No. of small saws, made from watch main-springs, hardly the thirtieth part of an inch wide, having a frame specially made for using them, although we did at first use a file carrier, such as is used for separating the molar teeth, but the saw is required in this to be too short, and it is usually too bulky and inconvenient. The saw should be some two or two and a half inches long. With this the crown may be very expeditiously cut off, even with the gum, leaving the stump concave, and requiring very little, if any filing. It is not half so disagreeable to the patient as the file, done in one fourth the time, and jarring the fang less; is by far less liable to subject the periosteum to inflammation. E. T.

